# Increasing Evidence That Irritable Bowel Syndrome and Functional Gastrointestinal Disorders Have a Microbial Pathogenesis

**DOI:** 10.3389/fcimb.2020.00468

**Published:** 2020-09-09

**Authors:** Caterina Carco, Wayne Young, Richard B. Gearry, Nicholas J. Talley, Warren C. McNabb, Nicole C. Roy

**Affiliations:** ^1^School of Food and Advanced Technology, Massey University, Palmerston North, New Zealand; ^2^Riddet Institute, Massey University, Palmerston North, New Zealand; ^3^Food Nutrition and Health Team, AgResearch Grasslands, Palmerston North, New Zealand; ^4^The High-Value Nutrition National Science Challenge, Auckland, New Zealand; ^5^Department of Medicine, University of Otago, Christchurch, New Zealand; ^6^Faculty of Health and Medicine, University of Newcastle, Callaghan, NSW, Australia; ^7^Liggins Institute, University of Auckland, Auckland, New Zealand; ^8^Department of Human Nutrition, University of Otago, Dunedin, New Zealand

**Keywords:** human microbiota, immunity, irritable bowel syndrome, functional gastrointestinal disorders, diet, visceral pain, motility, host-microbe interactions

## Abstract

The human gastrointestinal tract harbors most of the microbial cells inhabiting the body, collectively known as the microbiota. These microbes have several implications for the maintenance of structural integrity of the gastrointestinal mucosal barrier, immunomodulation, metabolism of nutrients, and protection against pathogens. Dysfunctions in these mechanisms are linked to a range of conditions in the gastrointestinal tract, including functional gastrointestinal disorders, ranging from irritable bowel syndrome, to functional constipation and functional diarrhea. Irritable bowel syndrome is characterized by chronic abdominal pain with changes in bowel habit in the absence of morphological changes. Despite the high prevalence of irritable bowel syndrome in the global population, the mechanisms responsible for this condition are poorly understood. Although alterations in the gastrointestinal microbiota, low-grade inflammation and immune activation have been implicated in the pathophysiology of functional gastrointestinal disorders, there is inconsistency between studies and a lack of consensus on what the exact role of the microbiota is, and how changes to it relate to these conditions. The complex interplay between host factors, such as microbial dysbiosis, immune activation, impaired epithelial barrier function and motility, and environmental factors, including diet, will be considered in this narrative review of the pathophysiology of functional gastrointestinal disorders.

## Background

In the human body there are about 39 trillion microbial cells (Sender et al., 2016), the majority of which inhabit the gastrointestinal (GI) tract, forming a dynamic ecological environment collectively known as the microbiota (Schulberg and De Cruz, 2016). The microbiota encompasses up to 500 transient and indigenous species, including bacteria, viruses, fungi and protozoa, and comprises up to 20 million genes (Sender et al., 2016).

The microbial ecosystem exists in a mutualistic relationship with its host and plays a crucial role in the maintenance of a healthy GI tract. The microbiota exerts important functions for the human organism, such as the extraction of energy from nutrients, metabolism of xenobiotics, modulation of motility and improved integrity of the epithelial barrier (Fava and Danese, 2011; Kashyap et al., 2013).

Therefore, the GI microbiota contributes to the beneficial effects of food beyond provision of nutrients (Louis et al., 2007). It is now accepted that its composition and function potentially contribute to the pathophysiology of functional GI disorders (FGIDs) (Enck et al., 2016). These conditions are classified by GI symptoms related to any combination of motility disturbance, visceral hypersensitivity, alterations of central nervous system processing, immunity and GI microbiota (Schmulson and Drossman, 2017). Irritable bowel syndrome (IBS) is the most common and best known of these disorders (Choung and Locke, 2011), characterized by abdominal pain associated with altered bowel movement and often bloating in the absence of morphological changes (Enck et al., 2016). However, the mechanisms responsible for FGIDs are poorly understood and there is a lack of consensus on what the exact role of the microbiota is, and how changes to it relate to these conditions.

The concept of the “brain in the gut” is not new (Alexander, 1934). The GI wall contains about 100 million nerve cells and more than 70% of the total immune system (Vighi et al., 2008). Microbial and dietary antigens interact with these pathways, aiding in inducing and maintaining homeostasis, while preserving responsiveness to pathogenic stimuli (Tlaskalová-Hogenová et al., 2011). This dynamic network, which involves the neuroendocrine, immune and metabolic pathways, is defined as the microbiota-gut-brain axis, and autonomously regulates many GI physiological functions, including motility, secretion, immunity and thereby inflammatory processes (Holzer et al., 2001). This finding has been highlighted in germ-free mice, which are characterized by a reduced surface area in the ileum (Abrams et al., 1963), shallower villous crypts (Thompson and Trexler, 1971), lower levels and activity of T and B cell subsets (Imaoka et al., 1996) and limited lymphatic tissue (Tlaskalová-Hogenová et al., 1983).

FGIDs represent a serious economic and social problem. They are a common cause of primary and secondary care consultations, are associated with increased rates of gastroenterological and non-gastroenterological investigations and treatments, and lead to significant morbidity and direct healthcare costs (Canavan et al., 2014; Tack et al., 2019). However, the indirect costs of education and work absenteeism and presenteeism, reduced social interactions and time away from usual activities are even greater (Zhang F. et al., 2016). At present, the management of FGIDs relies on the palliation of symptoms. The key to developing effective treatments is a better understanding the etiology and pathophysiology of these disorders.

Therefore, a complex interplay of several factors seem to underlie the pathophysiology of IBS, but a growing body of evidence supports the role of the GI microbiota and innate immune system alterations (Ford and Talley, 2011). This narrative review summarizes the current knowledge regarding the microbial and immunological mechanisms underlying the pathogenesis of IBS. A PubMed search of all available English-language articles to date was conducted, using the following search terms: “irritable bowel syndrome,” “functional gastrointestinal disorders,” “microbiota” or “microbiome,” “dysbiosis,” “low-grade inflammation,” “pathophysiology,” “immunity, “diet,” “visceral pain,” “motility” and “host-microbe interactions.” The search was extended by using the references of selected recent articles and systematic reviews or meta-analysis. Host factors, such as microbial dysbiosis, low-grade inflammation, altered epithelial barrier function and motility, as well as environmental factors, including diet, will be considered to help shed light on the emerging pathophysiology of FGIDs.

## Irritable Bowel Syndrome and Functional Gastrointestinal Disorders

IBS is a multifactorial condition characterized by chronic and relapsing abdominal pain and altered bowel habit. The symptoms of IBS can overlap with those of other FGIDs and it has been estimated that up to a third of patients with FGIDs have features of more than one, suggesting a common underlying etiology (Aziz et al., 2018). IBS has not been found to have a single etiological cause, but is likely to be the result of genetic, environmental and dietary factors. Diagnoses of FGIDs rely on symptom-based criteria (Heizer et al., 2009), including symptom severity and frequency (sporadic, daily) and stool characteristics (Talley, 2008). These characteristics allow for classification of patients with IBS into mutually-exclusive categories according to Rome IV criteria, depending on their predominant bowel habit: diarrhea-predominant (IBS-D), constipation-predominant (IBS-C), mixed diarrhea/constipation (IBS-M), and unclassified (IBS-U). Rome IV criteria provide parameters for the diagnosis of IBS based on abdominal pain and altered bowel habit in the absence of specific pathology (Schmulson and Drossman, 2017). However, bloating, passage of mucus and incomplete rectal evacuation, which are common and troublesome symptoms in people with IBS, are not included in the Rome criteria (Lacy and Patel, 2017). IBS subjects can be further classified as sporadic (nonspecific), post-infectious or inflammatory bowel disease-associated IBS. In contrast to sporadic IBS, post-infectious IBS occurs after an episode of infectious gastroenteritis (Sadeghi et al., 2019), and inflammatory bowel disease-associated IBS indicates IBS-like symptoms in patients with clinically quiescent inflammatory bowel diseases (Quigley, 2016). FGIDs also include functional constipation (FC) and functional diarrhea (FD) where there is a significant change in bowel habit but not abdominal pain, in the absence of alternative pathology.

**Figure 1:**
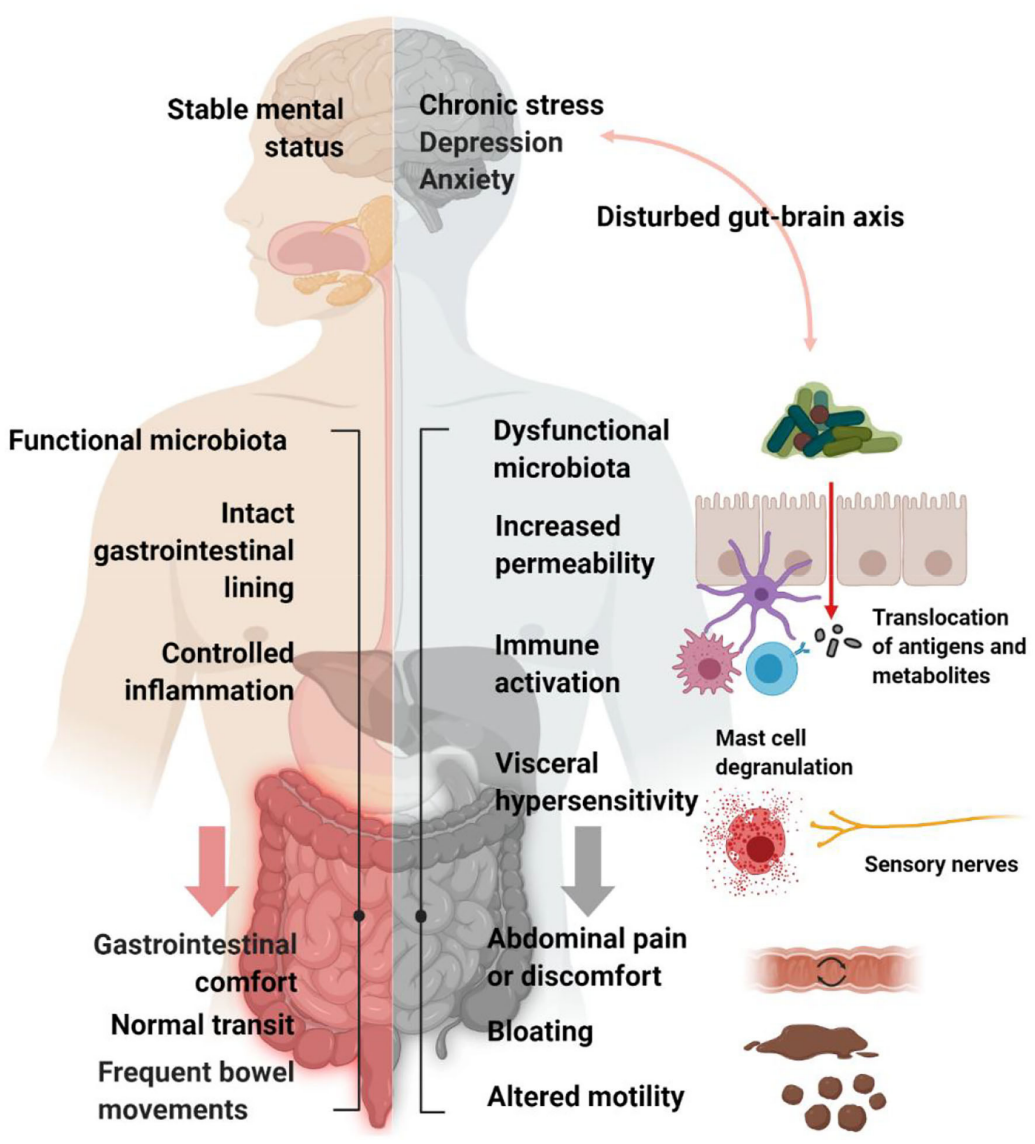
Schematic representation of IBS pathophysiology. Psychological, physiological and neuro-gastroenterological factors are thought to be involved in the generation of IBS symptoms, including bloating, abdominal pain and altered motility. Created with BioRender.com.

These heterogeneous conditions are also described as “disorders of gut-brain interaction,” as they can be classified as disorders that span both the GI and the neurological systems (Figure 1). People with these FGIDs have high rates of psychological comorbidity (Wu, 2012) and treatments aimed at stress and anxiety [e.g., hypnotherapy (Simon et al., 2019), cognitive behavioral therapy (Everitt et al., 2019), exercise (Zhou et al., 2019), and antidepressants Kulak-Bejda et al., 2017] can be effective treatments.

A number of proposed pathophysiological mechanisms for FGIDs are based in altered neuro-gastroenterology, including changes in GI motility and visceral afferent hypersensitivity. Visceral hypersensitivity tends to be more strongly associated with IBS than with FC or FD, although many subjects with FC report abdominal pain (Wong et al., 2010), yet IBS-C patients report a shorter colonic transit time (Ansari et al., 2010) and more severe symptoms of constipation compared to FC (Drossman, 2006). Furthermore, disorders of GI physiology, including mucosal permeability, bloating associated with discomfort and pain, immunity, and GI microbial dysbiosis, have also been shown to impact on psychological health (Sundin et al., 2014; Sinagra et al., 2020).

Since several conditions feature symptoms which may be confused with IBS, a clinical overlap between IBS and other IBS-like disorders has been proposed. In particular, the overlap between IBS and functional dyspepsia and gastroesophageal reflux disease, characterized by early satiety, postprandial fullness, epigastric pain, heartburn and regurgitation, is often associated with a more severe symptomatology (Jung et al., 2007; von Wulffen et al., 2019). IBS is also commonly associated with non-GI symptoms that are seen in other disorders, including fibromyalgia, chronic fatigue and temporomandibular joint disorder (Aaron et al., 2000). IBS was also observed in 33% of individuals reporting sleep disturbance (Vege et al., 2004), and in 48% of individuals with bladder pain (Kennedy et al., 2006).

Although not fatal and uncommonly requiring hospitalization, IBS is amongst the most frequent reasons for presentation to primary care. This leads to increased costs through consultations with health care practitioners, investigations for GI and non-GI disorders and subsequent treatments. Overall it is estimated that more than 40% of people worldwide suffer from FGIDs (Sperber et al., 2020). IBS affects 11% of the global adult population (Lovell and Ford, 2012; Enck et al., 2016), with a higher prevalence (60–75%) in women than men, especially for IBS-C (Jones et al., 2014). Sex hormones have been postulated to be responsible for this gender difference, because of their involvement in the stress response, colonic motility, epithelial barrier function, immune activation, and several regulatory mechanisms of the gut-brain axis (Kim and Kim, 2018). Sex hormones can also directly affect microbiota metabolism and composition through the estrogen receptor β (Menon et al., 2013). Alternatively, altered immune activation in IBS has been observed and, like in autoimmune diseases, it may account for a female predominance (Talley, 2020).

The severity of abdominal pain and the unpredictability of bowel function are the major factors lowering the quality of life of people with IBS, who report quality of life scores close to or lower than individuals with rheumatoid arthritis and dialysis-dependent kidney failure (Gralnek et al., 2000; Frank et al., 2002). Despite being so common and having such a significant impact on quality of life for so many, research into FGIDs such as IBS has been relatively underfunded. There is a large unmet need for people with FGIDs such as IBS. Understanding the etiology and pathophysiology promises an opportunity to develop new, effective and personalized treatments in addition to biomarkers for diagnosis, determining severity and treatment response.

## A Microbial Signature of IBS

In the GI tract, the most abundant phyla are Firmicutes and Bacteroidetes, but Actinobacteria, Proteobacteria, Verrucomicrobia and the less represented Fusobacteria, Tenericutes, Spirochaetes and Cyanobacteria are also present (Huse et al., 2008; Human Microbiome Project Consortium., 2012). The microbial composition changes across the different regions of the GI tract, with a predominance of Firmicutes in the proximal colon and Bacteroidetes in the distal colon (Sekirov et al., 2010).

The health-associated patterns of microbial colonization of the GI tract are difficult to define, as everyone can harbor functional and distinctive variants of microbial composition, reflecting early-life events such as mode of delivery, type of feeding and gender (Martin et al., 2016). Generally, a “healthy” microbial signature is characterized by a prevalence of Firmicutes and Bacteroidetes and a general lack of Proteobacteria (Hollister et al., 2014).

Despite inconsistencies between studies, some differences between a healthy and an IBS-related fecal microbiota have been observed. At the phylum level, a higher (Tana et al., 2010; Rajilić–Stojanović et al., 2011; Jeffery et al., 2012b; Tap et al., 2017) or lower (Jalanka-Tuovinen et al., 2014; Pozuelo et al., 2015) Firmicutes:Bacteroides ratio and differences in Actinobacteria and Proteobacteria prevalence have been observed in IBS (Labus et al., 2017).

At the genus level, IBS patients generally have increased *Ruminococcus* (Malinen et al., 2005; Lyra et al., 2009; Rajilić–Stojanović et al., 2011; Saulnier et al., 2011; Jeffery et al., 2019), *Clostridium, Coprococcus* and *Blautia* and reduced *Faecalibacterium* relative abundance (Rajilić–Stojanović et al., 2011; Carroll et al., 2012). These bacteria are thought to have a prominent role in carbohydrate metabolism in the colon.

Other alterations have been generally described in IBS, including an increase in the relative abundances of pathobionts, such as *Veillonella* (Malinen et al., 2005; Tana et al., 2010; Rigsbee et al., 2012), and *Enterobacteriaceae, Bacteroides* or a decrease in *Prevotella* (Rajilić–Stojanović et al., 2011) and *Desulfovibrionaceae* (Gobert et al., 2016). *Desulfovibrionaceae* include sulfur-reducing bacteria that compete with methanogens for hydrogen disposal in the human colon (Strocchi et al., 1994). Overall, differential relative abundance of taxa from the Bacteroidetes phylum and *Ruminococcaceae* or *Lachnospiraceae* families have been reported across studies (Rajilić–Stojanović et al., 2011; Jeffery et al., 2012b, 2019; Tap et al., 2017).

Previous studies have shown that methanogen relative abundance, exhaled methane level and symptom severity are negatively correlated with microbial richness, suggesting methane may contribute to slower GI motility and constipation (Sahakian et al., 2010; Falony et al., 2016; Tap et al., 2017). An increase in fecal *Methanobrevibacter smithii* and methane in breath from IBS-C patients has been reported (Ghoshal et al., 2016), as well as a positive association between *Methanobrevibacter* and stool firmness (Vandeputte et al., 2016). The elevated breath methane production in these individuals could alternatively reflect the outgrowth of “slow-growing” microbes, which are advantaged in conditions of slowed colonic transit and are resistant to the lack of water that characterize firmer stool (Quigley and Spiller, 2016). However, another study did not observe an association between breath methane production and constipation or colonic transit, although they reported an association between breath methane production and changes in fecal microbiota composition (Parthasarathy et al., 2016).

Other findings linked decreased levels of methanogens in feces to excess abdominal gas in IBS, suggesting that IBS patients compared to healthy subjects may lack some functions for hydrogen removal (Jalanka-Tuovinen et al., 2014; Pozuelo et al., 2015). Hydrogen accumulation has been linked to bloating and abdominal pain (Zhu et al., 2013). Hydrogen sulfide, deriving from the activity of sulfur-reducing bacteria, has been shown to modulate peripheral pain-related signals, as well as colonic motility (Jimenez et al., 2017).

**Figure 2:**
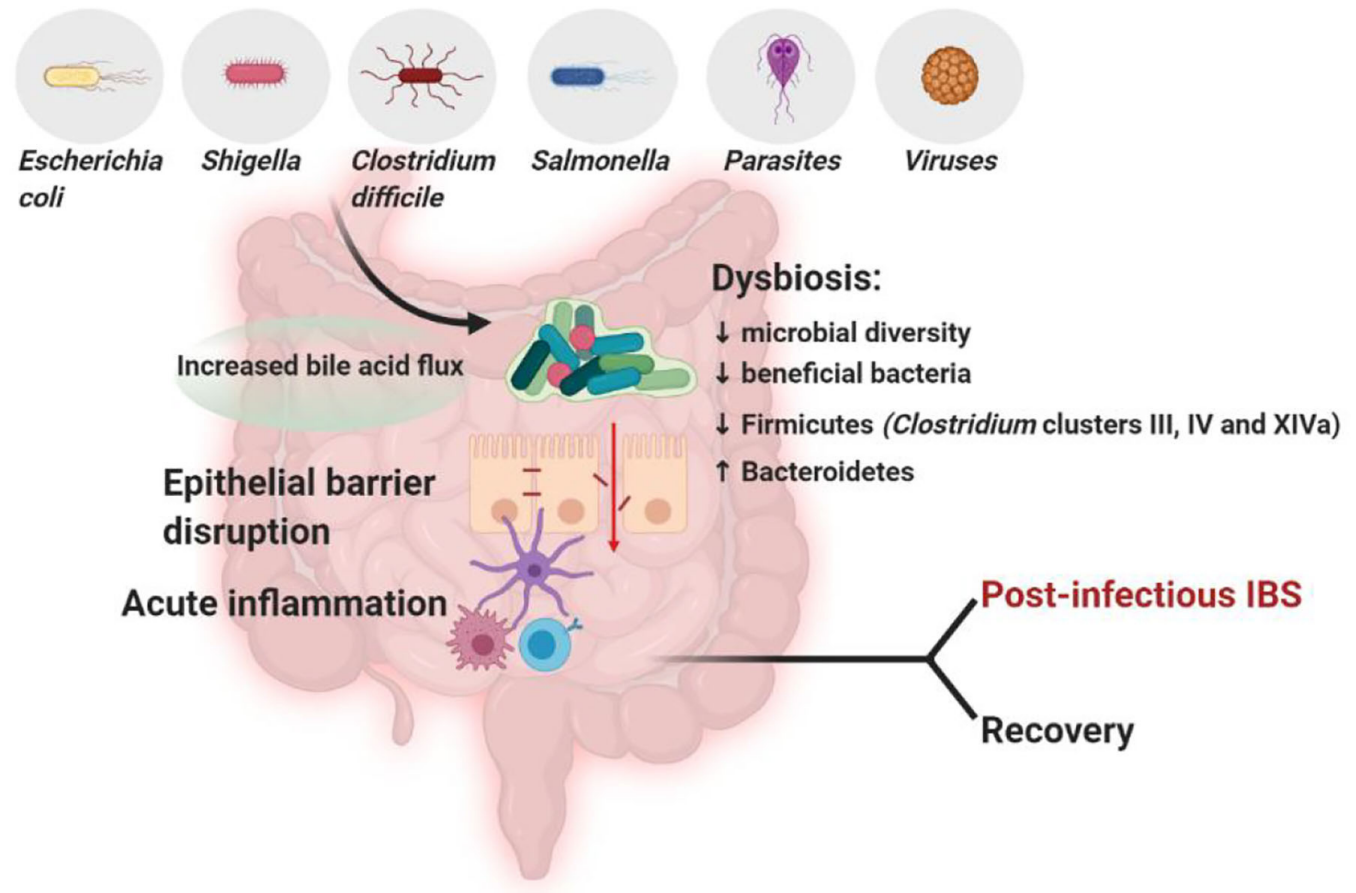
In subjects with post-infectious IBS, the infection by certain pathogens, such as *Clostridium difficile* (Wadhwa et al., 2016; Bassotti et al., 2018), *Salmonella* (McKendrick and Read, 1994), *Shigella* (Gwee et al., 1999; Wang et al., 2004) or *Escherichia coli* (Marshall et al., 2006) compromises the integrity of the epithelial barrier, triggers inflammation and decreases microbial diversity and beneficial bacteria, detrimentally affecting GI microbiota composition (Jalanka-Tuovinen et al., 2014). The microbiota composition in post-infectious IBS subjects differs from both IBS subjects and healthy controls, featuring an increase in Bacteroidetes, which are usually decreased in general IBS, and a decrease in Firmicutes, including *Clostridium* clusters III, IV and XIVa (Sundin et al., 2014). Created with BioRender.com.

The association between an altered microbiota and IBS is also supported by the fact that about 10% of the episodes of infectious gastroenteritis lead to the onset of IBS (Barbara et al., 2019) (Figure 2).

Several studies report discrepancies in fecal microbiota profiles between the IBS subtypes. Some studies report no differences in the composition of the microbial community between IBS-C and IBS-D (Pittayanon et al., 2019), while other studies associated different IBS subtypes with an individual microbial signature (Table 1).

**Table 1:** Main differences in fecal microbiota composition between IBS subtypes.

	**IBS-C**	**IBS-D**	**IBS-M**
Phylum	↑ Firmicutes ↑ Actinobacteria	↑ F/B ratio ↑ Proteobacteria ↓ Bacteroidetes ↓ Actinobacteria	↑ F/B ratio
Class	↑ Clostridia		
Order	↑ Clostridiales ↑ Coriobacteriales		
Family	↑*Incertae Sedis* XIII *↑ Lachnospiraceae* *↑ Ruminococcaceae* *↑ Rhodospirillaceae* *↑ Coriobacteriaceae*	↓*Erysipelotrichaceae* ↓*Ruminococcaceae* ↓*Porphyromonadaceae* ↓*Ruminococcaceae* *↓ Unknown Clostridiales* *↓Methanobacteriaceae* *↓ Incertae sedis* XIII	↓ *Erysipelotrichaceae* ↓*Ruminococcaceae* *↓ Incertae sedis* XIII *↓ Eubacteriaceae*
Genus	↓ *Roseburia* ↓*Bifidobacterium*	↓*Bifidobacterium* ↓*Lactobacillus*	
Species	↓*Eubacterium rectale* ↓*Eubacterium hallii* ↓*Anaerostipes caccae* ↑*Methanobrevibacter* smithii		

*F/B ratio, Firmicutes:Bacteroidetes ratio (Duan et al., 2019)*.

IBS-C usually features higher amounts of Firmicutes and a reduction in some lactate-producing and utilizing bacteria, such as *Bifidobacterium* and *Eubacterium hallii*/*Anaerostipes caccae*, respectively (Chassard et al., 2012). IBS-D is characterized by an overall reduction in microbial diversity, and an increase in potentially detrimental bacteria, such as Proteobacteria and lower numbers of Actinobacteria and Bacteroidetes, compared to IBS-C (Malinen et al., 2005; Carroll et al., 2012). Decreased relative abundances of *Bifidobacterium* in both fecal (Malinen et al., 2005; Kerckhoffs et al., 2009; Rajilić–Stojanović et al., 2011; Parkes et al., 2012) and mucosal samples (Kerckhoffs et al., 2009; Parkes et al., 2012), and *Lactobacillus* (Malinen et al., 2005) have been also described in IBS-D, although some studies reported the opposite findings (Tana et al., 2010; Carroll et al., 2011; Rigsbee et al., 2012; Labus et al., 2017). The reduction of *Bifidobacterium* and *Lactobacillus* is noteworthy, because of their capacity to exert bactericidal effects against pathogens and promote immune-tolerance through the production of metabolites, such as organic acids, including short-chain fatty acids (SCFAs) (Ma et al., 2018). These metabolites, mostly acetate, butyrate and propionate, represent the end-products of fermentation of non-digestible polysaccharides by the ileal and colonic microbiota (Havenaar, 2011). They are directly associated with host-microbe interactions through nutritional, regulatory and immunomodulatory functions.

Altered levels of SCFAs in feces appear to be associated with a different distribution of Clostridiales in IBS-C and -D, as well as with stool consistency (Gargari et al., 2018). The relative abundance of SCFA-producers, such as the Clostridiales order, the *Bifidobacterium* genus, and *Ruminococccaceae* and *Erysipelotrichaceae* families has been reported to be overall increased (Rajilić–Stojanović et al., 2011) or decreased (Pozuelo et al., 2015) in a IBS-related microbiota. *In vitro* studies previously demonstrated that SCFAs can lower the colonic pH (Duncan et al., 2009). Members from the Firmicutes phylum, particularly the *Clostridium* cluster XIVa, have been shown to more resistant to lower pH values compared to the Bacteroidetes.

Discrepancies on the relative abundance at lower taxonomic levels of beneficial bacteria and SCFA-producers may be explained by several factors, including differences in diet, study size, the predominance of IBS subtypes, IBS severity, as well as DNA extraction methods, analytic techniques or primers used for amplicon generation.

Instillation of SCFAs at high concentrations in the ileum may detrimentally result in increased ileal motility and abdominal pain in humans (Kamath et al., 1988) or promote visceral hypersensitivity in a rat model (Xu et al., 2013). These observations may be particularly relevant, since abnormal levels of SCFAs, visceral hypersensitivity and dysmotility are often observed in those with IBS.

On the other hand, a reduction in SCFA production or butyrate-producing bacteria relative abundance is also thought to have consequences on colonic inflammation and barrier defense. A lower relative abundance of butyrate-producing bacteria, such as *Roseburia* and *Eubacterium rectale*, was observed in subjects with IBS-C (Chassard et al., 2012), while the families *Erysipelotrichaceae* and *Ruminococcaceae* were found to be decreased in IBS-D and IBS-M (Pozuelo et al., 2015).

The relative abundance of specific genera also appears to positively correlate with IBS symptom severity. The composition linked to the IBS-D enterotype is the most different from “normal” in terms of composition and is associated with the most severe symptomatology (Tap et al., 2017). The immune profile associated with IBS-D has been also reported as different from the other subtypes and positively correlated with pain severity, dissatisfaction with bowel habits and overall GI symptoms (Choghakhori et al., 2017).

**Figure 3:**
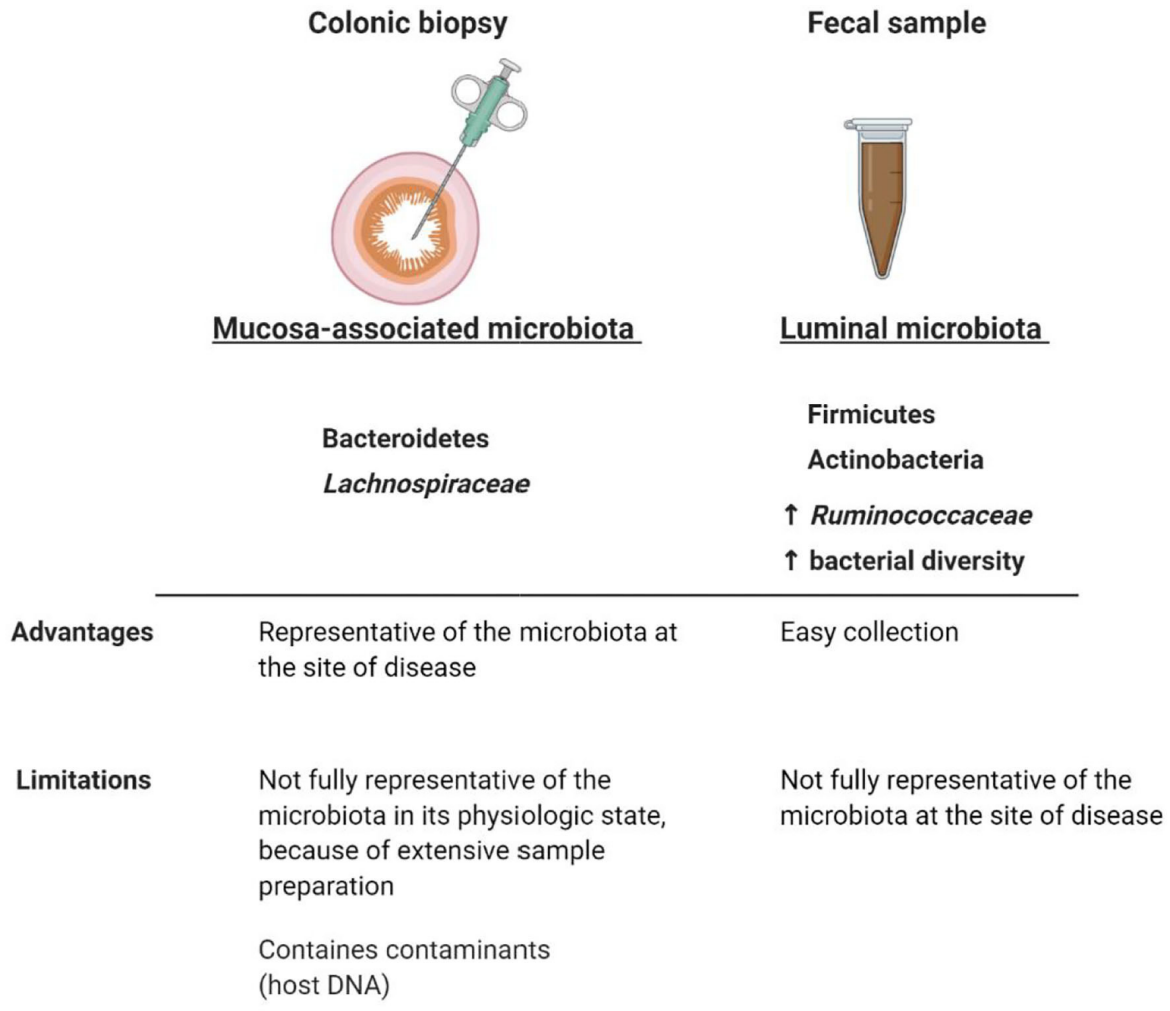
Comparison between mucosa-associated and luminal microbiota. Although luminal and colonic mucosal associated microbiota can potentially interplay with the immune system and therefore be involved in FGID symptomatology (Pittayanon et al., 2019), the fecal microbiota is not fully representative of the mucosal microbiota at the site of disease. Taxonomical and diversity differences between luminal and colonic mucosal microbiota highlight the importance of comparing the microbial composition in both niches, when analyzing the role of the GI microbiota in FGIDs. The colonic mucosa-associated microbiota seems to be predominantly characterized by Bacteroidetes (Rangel et al., 2015; Tap et al., 2017) and *Lachnospiraceae* (Hugerth et al., 2019), whereas the fecal microbiota by Firmicutes, Actinobacteria (Rangel et al., 2015; Tap et al., 2017), a higher relative abundance of *Ruminococcaceae* (Hugerth et al., 2019), and a higher bacterial diversity compared to the colonic mucosa-associated microbiota (Rangel et al., 2015). Microbial abnormalities in IBS subjects have been reported to be more pronounced in fecal samples than in colonic mucosal samples and the separation between mucosal and fecal microbiota composition was more distinct in IBS subjects than in healthy controls (Rangel et al., 2015). Whether IBS symptomatology is associated with taxonomical differences in the luminal and/or mucosal microbiota still remain to be determined. Created with BioRender.com.

The majority of studies investigating the GI microbiota from IBS subjects, analyzed only a single colonic niche (Malinen et al., 2005; Lyra et al., 2009; Saulnier et al., 2011; Carroll et al., 2012; Chassard et al., 2012; Jeffery et al., 2012b; Rigsbee et al., 2012; Jalanka-Tuovinen et al., 2014; Gobert et al., 2016), because of the convenience in collecting the fecal microbiota in comparison to the mucosa-associated microbiota (Figure 3). Fecal and mucosal microbiota have alternatively been reported to be structurally distinct but highly correlated (Tap et al., 2017), to discriminate between IBS-D subjects and healthy controls (Carroll et al., 2011), to discriminate only the subjects with severe IBS (Tap et al., 2017), or to not discriminate at all IBS subjects from healthy controls (Maharshak et al., 2018; Hugerth et al., 2019). Another study showed that the composition of the colonic mucosal microbiota could also separate patients with chronic constipation from controls with 94% accuracy (Parthasarathy et al., 2016).

The differences in microbial composition between IBS and healthy subjects as well as within IBS subtypes raise questions regarding which microbes are associated or not with IBS and which alteration between qualitative (dysbiosis) and quantitative (bacterial overgrowth) comes first in IBS etiology. The usefulness of describing the microbiota at higher taxonomic levels may be limited, since this may not provide meaningful information. New metagenomic tools allow an integrated analysis of taxonomic and predictive functional dynamics of the microbiota, providing improvements in genus-species analyses, more detailed insights into the effect of microbial metabolic pathways on crucial aspects of IBS pathogenesis, as well as of the potential host-microbiota interactions in health and disease. In addition, current techniques relying for example on 16S rRNA gene analysis, may also overlook potential pathogens, such as colonic spirochetes, which may be linked to symptoms of IBS, due to the incompatibility of standard primers (Thorell et al., 2019). Colonic spirochetosis has been associated with colonic eosinophilia and with non-constipating IBS (Walker et al., 2015).

Clinical evidence also supports the involvement of the GI microbiota in IBS pathogenesis. Rifaximin, a non-systemic antibiotic which is efficacious for the treatment of IBS-D (Lembo et al., 2016), showed a largely transient effect across a broad range of stool microbes, such as *Peptostreptococcaceae, Verrucomicrobiaceae* and *Enterobacteriaceae*, in a randomized, double-blind, placebo-controlled study with IBS-D subjects (Fodor et al., 2019). Fecal microbiota transplantation with the aim of restoring the GI microbiota of IBS subjects to a healthy status have also demonstrated positive outcomes depending on the mode of delivery (Mazzawi et al., 2018, 2019; Ianiro et al., 2019), although conflicting results have been reported (Halkjaer et al., 2018; Johnsen et al., 2018).

## Microbial Modulation of Immunity and Homeostasis

**Figure 4:**
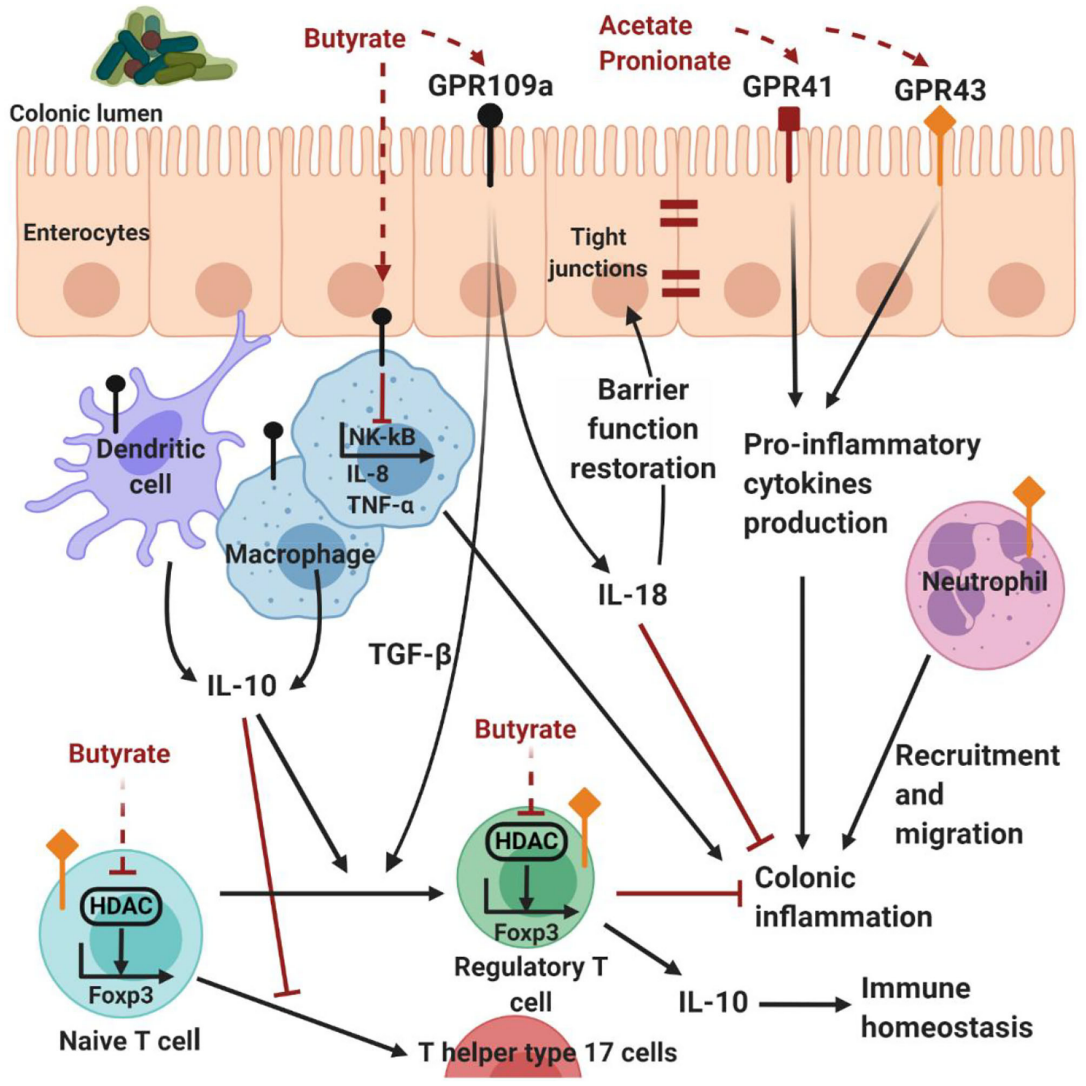
Host-microbe interactions mediated by SCFAs. G-protein-coupled receptor expressed on intestinal epithelial and immune cells are activated by SCFAs. In particular, acetate and propionate are the most efficient agonists for GPR43 and GPR43, followed by butyrate and then other SCFAs (Kim et al., 2013). Propionate agonizes GPR43 on colonic regulatory T cells to inhibit HDAC function and enhance FOXP3 expression, thereby promoting regulatory T cell differentiation and IL-10 production. Although acetate is a potent GPR43 ligand, and mediates colonic regulatory T cells accumulation, it is not clear whether this is through this receptor (Kim et al., 2013). Butyrate has similar effects by either stimulating dendritic cells and macrophages to produce IL-10, or directly acting on naive T cells, inhibiting the activity of HDAC on the Foxp3 gene, inducing naive CD4+ T cells differentiation and regulatory T cell expansion (Kim et al., 2013). Butyrate can induce the production of TGF-β and cytoprotective IL-18 by the enterocytes through the activation of GPR109A. In addition, butyrate can inhibit NF-κB signaling, reducing the expression of pro-inflammatory IL-8 and TNF-α (Kim et al., 2013). On the other hand, SCFAs can mediate protective immunity, activating GPR41 and GPR43 on GI epithelial cells and resulting in the production of pro-inflammatory chemokines and cytokines (Kim et al., 2013). Therefore, SCFAs contribute to the maintenance of intestinal homeostasis through multiple mechanisms. Created with BioRender.com.

Several studies highlight the immunological and regulatory effects of microbially-derived molecules, such as SCFAs, as an important link between the GI microbiota and the host. SCFAs are well known for modulating inflammatory responses from innate immune cells through different signaling pathways. For instance, butyrate can act as an inhibitor of histone deacetylases (HDAC), regulatory proteins acting on the epigenome through chromatin-remodeling changes (Arpaia et al., 2013). Alternatively, SCFAs can interact with G-protein-coupled receptor (GPR)41, GPR109A and GPR43, which are abundantly expressed on intestinal epithelial cells, monocytes and neutrophils, to decrease pro-inflammatory cytokine and dampen inflammatory responses (Masui et al., 2013; D'Souza et al., 2017). GPR109A, a receptor for niacin, is agonized by butyrate in the colon, promoting regulatory T cells differentiation, interleukin (IL)-10 and IL-18 expression in the colonic epithelium (Singh et al., 2014). IL-18 can have a dual role in inflammation and, in this case, it promotes epithelial restoration and inflammation recession (Pu et al., 2019). On the other hand, SCFAs can mediate protective immunity in particular conditions. For example, SCFAs activate GPR41 and GPR43 on GI epithelial cells, resulting in the rapid production of pro-inflammatory chemokines and cytokines (Kim et al., 2013) (Figure 4).

In addition, SCFAs are well known for modulating also immune cell chemotaxis, phagocytosis, reactive oxygen species release and reduction of NF-κB activity. The effect on NF-κB signaling, assessed on the human colon adenocarcinoma cell line, Colo320DM, has been shown by all three major SCFAs, in order of potency being butyrate>propionate>acetate (Tedelind et al., 2007). In particular, butyrate has been shown to inhibit the production of pro-inflammatory IL-8 and tumor necrosis factor (TNF)-α by macrophages *in vitro* (Park et al., 2007) and *in vivo* (Sokol et al., 2008).

However, despite the potential relevance of abnormal levels of colonic SCFAs in IBS pathophysiology, findings are inconsistent and often conflicting between studies. In subjects with IBS, altered levels of fecal SCFAs have been reported either increased or decreased. However, a recent meta-analysis attempting to clarify these alterations, identified an overall reduction of butyrate and propionate in fecal samples of IBS-C subjects and higher levels of butyrate in fecal samples of IBS-D subjects, when compared to healthy controls (Sun et al., 2019).

These findings support the role of the GI microbiota in the modulation of the immune responses from the host. However, this relationship exists in a mutual interaction where the adaptive and innate immune systems are likely to shape the composition of the microbiota in return. This hypothesis is supported by several arguments, for example, in mice the absence of the myeloid differentiation primary response 88, an adapter protein involved in toll-like receptor (TLR) signaling leads to Bacteroidetes overgrowth (Wen et al., 2008). In addition, the risk of developing IBS after an episode of gastroenteritis (Spiller et al., 2000) suggests that the activation of the immune system by infectious triggers including bacteria, viruses or parasites, could impact the composition and function of the microbial community.

Further evidence of these mutual microbe-immune interactions in IBS is the presence of antibodies against the pro-inflammatory bacterial protein flagellin (Schoepfer et al., 2008). Flagellin is capable of inducing antibody responses through TLR5 (Lopez-Yglesias et al., 2014), and an increased abundance of flagellin-producing species belonging to *Clostridium* cluster XIVa has been reported in IBS subjects (Salonen et al., 2010; Jeffery et al., 2012a). In particular, the mucin degrader *Ruminococcus torques* is known to produce flagellin proteins (Lyra et al., 2009) and is also frequently associated with IBS (Malinen et al., 2010). Because of these functional features, this species has been proposed as a potential player in the modulation of the low-level inflammatory responses at the mucosal surface.

Different species of commensals have been reported to induce specific effects on the host immune responses in health and disease. *Bacteroides fragilis* was demonstrated to have a protective role by inducing the proliferation of IL-10 producing-regulatory T cells, through the expression of the surface factor polysaccharide A (Round and Mazmanian, 2010).

Similarly, IL-10 release is also promoted by several Clostridia strains. Seventeen bacterial strains isolated from a healthy human fecal sample and falling within the *Clostridium* clusters IV, XIVa and XVIII have been demonstrated to increase the number and function of colonic regulatory T cells in colonized rodents (Atarashi et al., 2013). Moreover, since the Clostridia class is thought to colonize the area surrounding the colonic mucosa and includes several major butyrate-producers (Lopetuso et al., 2013), it is likely that taxa belonging to this class have a crucial impact on the host immune system.

Several species from the Clostridia class are also able to generate biologically active catecholamines, including the neurotransmitters norepinephrine and dopamine, as demonstrated in gnotobiotic and germ-free mice (Asano et al., 2012). Therefore, Clostridia seem to be particularly involved in IBS pathophysiology, because of their crucial role not only in GI immune homeostasis but also in the gut-brain axis.

The high co-morbidity between FGIDs and stress-related symptoms represents further evidence of the involvement of the gut-brain axis in IBS (Mayer et al., 2014). Animal models of stress-related disorders showed critical changes in fecal (Bharwani et al., 2016) and mucosal (Galley et al., 2014) microbial composition, metabolites (Aoki-Yoshida et al., 2016), immune gene expression in the terminal ileum, as well as in serum cytokine concentration (Aoki-Yoshida et al., 2016; Bharwani et al., 2016). This suggests that the microbiota is sensitive to stress exposure and highlights the importance of analyzing the microbiota community composition by microbial niche. Maes et al. were the first to demonstrate that psychological stress in humans induces inflammatory responses with increased production of the pro-inflammatory cytokines interferon (IFN)-γ, TNFα and IL-6 (Maes et al., 1998). In addition, stress-induced mediators, such as the corticotropin-releasing factor, increased macromolecular permeability in the healthy human colon via corticotropin-releasing factor receptor on subepithelial mast cells (Wallon et al., 2008). These findings may be relevant in the context of FGIDs, whose course is likely to be affected by persistent stress.

Crucial host-microbiota-immune interactions in the GI tract and in the central nervous system can also be affected by the availability of the essential amino acid tryptophan (Marsland, 2016; Rothhammer et al., 2016), and by the metabolites deriving from bacterial tryptophan metabolism (indole, indolic acid derivatives, skatole, and tryptamine). In IBS, increased tryptophan metabolism is associated with low-grade inflammation and microbiota alterations (Clarke et al., 2009). Tryptophan is also crucially involved in several other microbiota-mediated interactions in the GI tract, such as secretory and sensory reflexes, peristalsis and the serotonin pathway (Keszthelyi et al., 2009). A link between the microbiota and the tryptophan metabolism has been demonstrated in germ-free mice, which exhibit abnormal levels of serotonin in the colon but not in the small intestine (Yano et al., 2015).

In the body, the majority of serotonin, a crucial neurotransmitter and regulatory factor, is derived from the hydroxylation of L-tryptophan by the tryptophan hydroxylase 1 enzyme, expressed in intestinal enterochromaffin cells. Mucosal biopsies from individuals with IBS showed reduced mRNA expression levels of tryptophan hydroxylase 1 (Kerckhoffs et al., 2012). Therefore, dysregulation of the tryptophan pathway, which may affect mood and cognition, colonic motility and visceral hypersensitivity (O'Mahony et al., 2015), may be related to IBS pathogenesis. Similarly, a reduced serotonin reuptake and an impaired serotonin release have been reported respectively in subjects with IBS-D and IBS-C (Atkinson et al., 2006). In this regard, tegaserod, which is used to treat IBS-C, and alosetron, which is used to treat IBS-D, respectively stimulate and block the serotonin 5HT4 and 5HT 3 receptor (Binienda et al., 2018). This reflects the complexity of the interactions underlying abnormal colonic motility.

Overall, the unavoidable interaction between the GI microbiota and the immune system could potentially be involved in the low-grade chronic inflammation often observed in individuals with IBS regardless of subtypes. Inflammation may potentially underlie most of the pathways involved in IBS symptom generation, including visceral hypersensitivity (Klooker et al., 2010), abdominal pain (Barbara et al., 2004) and increased permeability (Wallon et al., 2008). However, the mechanisms behind the connection between stress, inflammation and colonic mucosal barrier function are largely unknown.

## Microbial Regulation of Epithelial Barrier Function in the GI Tract

In a healthy GI tract, the direct contact between the microbiota and the rest of the host is prevented by the mucosal barrier, that, together with the mucus layer, represents a “shield” against pathogens. The mucosal barrier also includes the mucosal immune system and the enteric nervous system (Kelly et al., 2015).

Mucins are highly glycosylated macromolecule components of the mucus barrier. They represent an alternative substrate to dietary polysaccharides for mucin-degrading bacteria, such as *R*. *torques* and *Akkermansia muciniphila* (Tailford et al., 2015). An abnormal increase in these species (such as through dietary restriction) may reduce mucus layer thickness, possibly contributing to impaired mucus barrier function, increased pathogen susceptibility and inflammatory conditions (Pelaseyed et al., 2014). An altered relative abundance of mucin-degraders may otherwise reflect changes in mucus shedding in subjects with IBS-D, resulting in mucous discharge in their stool.

The metabolism of sulfated mucins by mucin-degrading bacteria represents a source of sulfate, which can be subsequently reduced to hydrogen sulfide (Gibson et al., 1993). High concentrations of hydrogen sulfide have been demonstrated to induce oxidative stress, to impair cellular respiration and adenosine triphosphate production (Cooper and Brown, 2008) and to inhibit butyrate oxidation by colonocytes *in vivo* (Jorgensen and Mortensen, 2001) and *in vitro* (Roediger et al., 1993). Colonocytes are therefore deprived of their main sources of energy. Oxidative stress and energy starvation may result in colonocyte death, weakening of the epithelial barrier and direct contact of commensals with the mucosal immune system (Jorgensen and Mortensen, 2001). Therefore, increased levels of hydrogen sulfide, in conjunction with increased microbial nitric oxygen production and decreased mucosal sulfide detoxification, have been shown to damage the colonic epithelium and contribute to mucosal inflammation (Roediger and Babidge, 2000).

The GI microbiota can also directly control epithelial permeability by upregulating tight junction (TJ) proteins in both normal and pathological conditions (Ewaschuk et al., 2008; Anderson et al., 2010; Karczewski et al., 2010). Given this crucial role played by the commensals in the maintenance of epithelial barrier integrity, alterations in this community may be relevant for the increased permeability often seen in IBS-D (Dunlop et al., 2006; Hou et al., 2017). In particular, biopsies from subjects with IBS-D showed a reduced expression of occludin (Coeffier et al., 2010) and claudin-1 in the colonic mucosa (Bertiaux-Vandaele et al., 2011) and a disrupted apical junctional complex integrity in the jejunal mucosa (Martínez C. et al., 2013).

Alterations of TJ proteins in IBS have been also associated with visceral hypersensitivity, abdominal pain (Piche et al., 2009; Bertiaux-Vandaele et al., 2011) and mast cell activation (Martínez C. et al., 2013). The increased GI permeability may result in the translocation of bacteria and their products through the barrier, influencing local and systemic immune responses and contributing to the low-grade inflammation in IBS (Kelly et al., 2015). Pro-inflammatory cytokines such as IFN-γ, TNF-α, IL-4, IL-12 and IL-1β also contribute to TJ disruption and increased paracellular permeability (Suenaert et al., 2002). Hypersensitivity and symptom severity have been observed to be increased in IBS-D patients with increased GI permeability, in comparison to healthy controls and IBS-D subjects with normal permeability (Zhou et al., 2009).

A subtype-specific increase of mucosal mast cell mediators, such as serine proteases and tryptases, in subjects with IBS-D may be responsible for the observed increased colonic permeability (Lee et al., 2010; Wilcz et al., 2011). In addition, an *in vitro* study demonstrated that plasma lipopolysaccharides and tryptase levels were increased in IBS-D, but not in IBS-C (Ludidi et al., 2015). The same study also showed an increased permeability when Caco-2 cells were exposed to plasma from IBS-D and IBS-C subjects, with a higher effect for IBS-D in comparison to IBS-C. In addition, IBS-D patients show distinctive transcription patterns regarding epithelial permeability, mast cell activity and TJ expression; for example occludens mRNA expression has been observed to be inversely correlated with the mRNA expression of tryptase (Martinez et al., 2012).

*In vitro* studies with Caco-2 monolayers (Piche et al., 2009) or murine tissues incubated with colonic (Cenac et al., 2007) or fecal (Gecse et al., 2008) supernatants from IBS subjects support the correlation between decreased epithelial barrier function, zonula occludens-1 mRNA expression, inflammation and pain severity. Intestinal permeability in IBS may be possibly ameliorated by the positive effect exerted by lactic-acid bacteria on TJ proteins. Indeed, a probiotic cocktail including *Streptococcus thermophilus, Lactobacillus* spp. and *Bifidobacterium longum* has been demonstrated to improve mucosal barrier function in subjects with IBS-D (Zeng et al., 2008). Probiotics are live microorganisms that may be beneficial for conditions featuring dysbiosis, such as IBS. Recent systematic reviews and meta-analyses reported contrasting results (Ford et al., 2018a,b), but suggest that probiotics as a class, have very limited but beneficial effect over placebo on general IBS symptoms, such as bloating and flatulence (Ford et al., 2018b).

In conclusion, increased GI permeability, which seems to be a prevalent feature of IBS-D, may trigger low-grade GI and systemic inflammation and correlates with symptom severity. The molecular mechanisms responsible for increased GI permeability in FGIDs are still poorly understood, but represent potential therapeutic and discriminating targets for IBS-D from other IBS subtypes and health. Although there is a lack of concrete evidence to confirm these interactions, hypersensitivity to certain food have been identified as one of the possible causes for the increased epithelial barrier permeability, visceral hypersensitivity and inflammation in up to 65% of IBS subjects (Simrén et al., 2001).

## The Link Between Dietary Components and Functional Gastrointestinal Disorders

A growing body of evidence supports the role of dietary macronutrients (carbohydrates, proteins and lipids) in inducing shifts in the GI microbiota, influencing host metabolic and immune markers (Shibata et al., 2017). Several molecules, either coming directly from food or released by commensals are likely to influence the activity of the immune system (Shibata et al., 2017).

Diet has been recognized to be involved in the predisposition or exacerbation of IBS, as up to 65% subjects with IBS report food to play a crucial role in their symptoms (Böhn et al., 2013). Three mechanisms have been proposed to explain the dietary intolerances in individuals with IBS: hypersensitivity to specific foods; hypersensitivity to food chemicals and luminal distension.

Food hypersensitivity may involve immunoglobulin E-mediated (atopic) or non-immunoglobulin E-mediated (non-atopic) reactions. Acute-phase immunoglobulin E-mediated hypersensitivity results in the activation of mast cells, eosinophils, and other immune cells and the release of molecules (histamine, leukotrienes) involved in GI symptom generation (Portincasa et al., 2017). Recent studies did not observe increased levels of immunoglobulin E in IBS subjects (Zar et al., 2005) nor correlated increased serum immunoglobulin E with IBS symptom severity (Nybacka et al., 2018), rectal eosinophilia (Akkuş et al., 2019), or colonic mast cell and eosinophil activation in IBS subjects (Bischoff et al., 1997). Finally, a recent study on IBS subjects showed that more than 50% of patients could have a response to specific foods, characterized by eosinophil activation but which was not associated with immunoglobulin E (Fritscher-Ravens et al., 2019). Therefore, although atopic reactions to specific foods are common in patients with IBS, the association with IBS pathogenesis is not supported in literature and immunoglobulin E-mediated food hypersensitivity in IBS is rare (Crowe, 2019).

There is increasing evidence that immunoglobulin G-mediated food hyperreactivity may play a role in IBS symptom generation, but results remain contradictory. Recent studies found elevated food-specific immunoglobulin G levels in IBS subjects in comparison to controls (Zar et al., 2005; Lee and Lee, 2017; Karakula-Juchnowicz et al., 2018). In a randomized controlled trial, IBS subjects excluded from their diet the foods responsible for their increased immunoglobulin G levels. After 3 months, the dietary exclusion resulted in a reduction of symptom severity, suggesting that food elimination based on immunoglobulin levels may be promising for the reduction of IBS symptoms (Atkinson et al., 2004). Notably, the 87% of the IBS subjects from this study reported symptomatic reactions to yeast, but previous studies with a similar number of participants observed lower percentages [5% Nanda et al., 1989 and 12% Hunter, 1985] of IBS patients indicating yeast as an offending food. Therefore, these discrepancies suggest that increased levels of immunoglobulin G to a specific food may not be necessarily linked to IBS symptom generation. Other findings confirmed that immunoglobulin G-mediated hypersensitivity to yeast or other specific foods in IBS is unlikely, as no differences were found in immunoglobulin G levels between IBS subjects and controls (Ligaarden et al., 2012). Moreover, either low or high immunoglobulin G levels were associated with more severe symptomatology (Ligaarden et al., 2012). Therefore, an increase production of immunoglobulin G is more likely to reflect a physiological response to diet rather than a pathological reaction from the GI immune system.

Secondly, food bioactive chemicals, such as salicylates, (contained for example in almonds, apples, berries.), or related organic or inorganic acids, have the potential to trigger a non-specific antigen-induced pseudo-allergic hypersensitivity reaction, causing the release of cysteinyl leukotrienes (Raithel et al., 2005). Cysteinyl leukotrienes are pro-inflammatory lipid mediators deriving from arachidonic acid which increase smooth muscle contraction and vascular permeability (Raithel et al., 2005), resulting in nausea, bloating, diarrhea or visceral hypersensitivity. Although salicylate sensitivity has been suggested to affect 2–7 % of individuals with inflammatory bowel diseases (Raithel et al., 2005), there is still a lack of concrete evidence linking salicylate sensitivity to FGIDs. In a survey of 643 subjects with IBS, 12% reported their symptoms to be associated with the combined use of analgesics, including the salicylate aspirin (Locke et al., 2000). However, the study also showed that these individuals were intolerant to a high number of foods, which could be associated with the reported symptoms.

**Figure 5:**
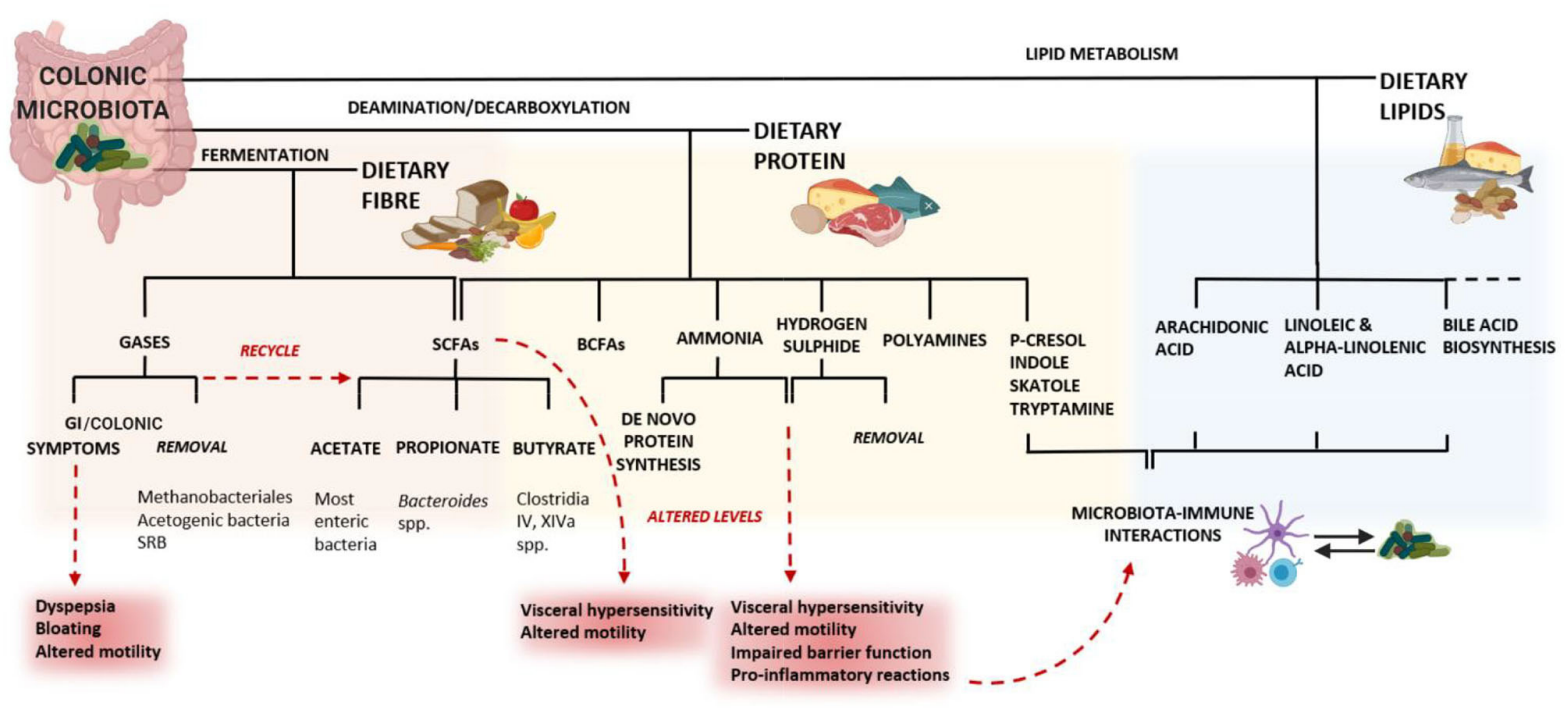
The consequences of diet on a dysbiotic microbiota may lead to altered levels of these metabolites, resulting in GI symptoms. In the colon, the fermentation of dietary fiber results in changes in the microbiota composition, supporting the growth of beneficial bacteria. Consequently, the microbiota generates gases, SCFAs and other metabolites. The microbial metabolism of lipids entering the colon is involved in several important pathways for the host. The families *Erysipelotrichaceae* and *Coriobacteriaceae* also play an important role in the conversion of cholesterol-derived metabolites, such as bile salts and steroids (Martínez I. et al., 2013). Altered bile acid metabolism has been associated with chronic inflammation in the colon (Devkota et al., 2012) and microbiota-derived bile acid metabolites have the potential to affect both host metabolism and immune responses (Alimov et al., 2019). The microbiota-mediated protein metabolism is largely affected by the proteolytic activity of amino acid-fermenting bacteria, mainly *Clostridia* and *Peptostreptococcus*, but also *Bacteroides* spp., *Propionibacterium, Fusobacterium* spp., *Streptococcus, Lactobacillus, Veillonella* spp., *Selenomonas ruminantium* and *Megasphaera elsdenii*are (Yang and Yu, 2018). The microbial catabolism of amino acids occurs mostly through deamination and decarboxylation (Bertrand et al., 2014) and can generate immuno-modulatory molecules and neurotransmitters (like catecholamines) that have effects on both the immune and the nervous system. For example, the microbial glutamate decarboxylases convert glutamate into gamma-aminobutyric acid, which has immunomodulatory effects in the GI tract (Baj et al., 2019). Histamine, derived from the bacterial decarboxylation of L-histidine, can inhibit the release of pro-inflammatory cytokines via the histamine type 2 receptor on epithelial cells (Thomas et al., 2012). Hydrogen sulfide is thought to be responsible for an increased visceral hypersensitivity related to colonic distension, for altered colonic motility (Tsubota-Matsunami et al., 2012) and other deleterious effect on the colonic epithelium (Jorgensen and Mortensen, 2001). SRB: sulfate-reducing bacteria; BCFAs: branched-chain fatty acids. Created with BioRender.com.

In this regard, the third mechanism involves a group of food components comprising a category of nutrients defined as fermentable oligosaccharides, disaccharides, monosaccharides and polyols (FODMAPs), which are short-chain, soluble, highly fermentable carbohydrates. Their fermentative properties make FODMAPs closely linked to symptoms generation in IBS (Figure 5), increasing the stool bulk with water and fermentation by-products (gas and SCFAs), often resulting in luminal distension, abdominal pain and bloating (Böhn et al., 2013).

A diet low in FODMAPs is very restrictive and although long-term restrictive diets seem to still allow for an adequate nutrients intake (O'Keeffe et al., 2018), they may decrease the absolute and relative microbial load and diversity. This can potentially lead to detrimental effects on the colonic environment and microbiota (Halmos et al., 2015).

FODMAPs appear to be the preferred fermentation substrate for the Clostridia class (Flint et al., 2012), so their relative abundance and their functional characteristics have been proposed to play a role IBS symptom generation. Because of their ability to influence the microbiota composition, fermentable carbohydrates (e.g., fiber) are the most investigated dietary category in the context of IBS (Martínez et al., 2010). Primary fiber-fermenters include *Ruminococcus bromii, Roseburia* and *Eubacterium rectale* (Walker et al., 2011; Martínez C. et al., 2013), which generate byproducts that are more easily utilized by other species, contributing to bacterial cross-feeding.

The scientific evidence of the use of fiber and bulking agents to possibly improve IBS symptoms has been reviewed in several meta-analyses, but the benefits seem to be too sparse to draw firm conclusions (Lesbros-Pantoflickova et al., 2004; Ford et al., 2008). Soluble fiber supplementation may ameliorate constipation in IBS, but symptoms like bloating and abdominal pain may not improve or even worsen with some types of fiber, such as wheat corn and bran (Bijkerk et al., 2004).

Dietary fiber can act as a prebiotic, affecting the composition of the colonic microbiota, promoting the growth of beneficial bacteria, such as *Lactobacillus* and *Bifidobacterium*, and increasing the production of SCFAs, which are important in the maintenance of intestinal homeostasis (Maslowski and Mackay, 2011). Furthermore, dietary fiber can also stimulate mucus production and secretion by the colonic epithelium (McRorie and McKeown, 2017).

On the other hand, the consumption of diets rich in saturated fats of animal origin has been associated with low-grade inflammation in the GI tract, through the activation of TLR-dependent signaling by microbial factors (Kim et al., 2012; Caesar et al., 2015). The host lipid metabolism has been often associated with the microbiota community composition, and particularly with the families *Erysipelotrichaceae* and *Coriobacteriaceae*. Some members of the *Coriobacteriaceae* are thought to be involved in metabolic disorders and FGIDs, and are therefore considered as fat-induced pathobionts (i.e., potentially pathogenic symbionts of the microbiota) (Clavel et al., 2014). Similarly, the relative abundance of *Erysipelotrichaceae* seem to be linked to diets high in fats and to play a role in host lipid metabolism (Harris et al., 2014) as well as in colonic inflammation. Indeed, some members of this bacterial family are coated with immunoglobulin A and therefore, highly immunogenic (Palm et al., 2014). Overall, it is unclear if *Erysipelotrichaceae* may play a role in the development of colonic inflammation or if their relative abundance is reflecting more the dietary and/or the lipid and cholesterol status of the host.

A high intake of dietary protein, specifically animal-based proteins, has been implicated in the pathogenesis of IBS through multiple mechanisms (Kakodkar and Mutlu, 2017). An excessive microbial fermentation of protein results in the release of toxic end-products, such as ammonia, phenols, branched-chain fatty acids, and hydrogen sulfide. *Clostridium* spp. have long been considered as major producers of ammonia from protein fermentation (Vince and Burridge, 1980), which can impair colonic barrier function (Lin and Visek, 1991) and stimulate the release of pro-inflammatory cytokines (Pieper et al., 2012). This may explain the fact that many IBS subjects report foods rich in animal protein, including meat, fish and eggs, to induce GI symptoms (Hayes et al., 2014).

Hydrogen sulfide, another end-product of protein fermentation, is produced by the microbiota mostly through the degradation of the sulfur-containing amino acid cysteine. *Fusobacterium* spp., which is known to generate cysteine through the cysteine desulfydrase activity, has been associated with impaired colonic function in IBS or inflammatory bowel diseases (Strauss et al., 2011). Although high concentrations of hydrogen sulfide can be detrimental for the colonic epithelium, hydrogen sulfide at low concentrations was demonstrated to maintain the integrity of the mucus layer and to ameliorate mucosal inflammation (Wallace et al., 2018). Given the fact that the colonic microbiota generates much more hydrogen sulfide from cysteine than the colonic epithelial cells, it has been suggested that hydrogen sulfide exerts a protective effect when produced from endogenous metabolism but can be deleterious when generated at high concentrations by colonic microbes (Blachier et al., 2019).

## Biomarkers Toward an Immune Signature in Functional Gastrointestinal Disorders

Understanding the mechanisms underlying host-microbe interactions and symptoms pathophysiology will likely improve the current knowledge of pathways involved and the predictive value of IBS biomarkers. Biomarkers can be measured in blood, fecal, urine or breath samples, to potentially discriminate IBS from other GI disorders or from health, and more importantly within the IBS subtypes and to characterize improvements in well-being and quality of life of IBS subjects.

General observations in IBS vs. health include differences in microbial composition, immune profile, GI motor and sensory function, pain perception, serotonin metabolism, and the expression of genes involved in immune activation (Camilleri et al., 2017). Differences in fecal bile acids and fecal fat also successfully discriminated between IBS-D and IBS-C (Vijayvargiya et al., 2019) and fasting serum C4 (7a-hydroxy-4-cholesten-3-one) and fibroblast growth factor 19 showed good specificity to exclude the diagnosis of bile acid diarrhea in IBS-D and FD (Vijayvargiya et al., 2017).

Some of the parameters that have been studied include biomarkers of GI and immune function and biomarkers of GI microbiota (Bischoff, 2011; Hyland et al., 2014). In 2009, Lembo et al. reported 10 “first-generation” serum biomarkers with high specificity (88%), although the sensitivity was poor (50%) (Lembo et al., 2009). However, reflecting the complex pathophysiology, the utility increased when the panel was expanded to 34 serological and gene expression markers to discriminate IBS from healthy controls (Jones et al., 2014). Subsequently, other studies combined plasma and fecal biomarkers associated with different parameters of GI function, to reflect the multifactorial nature of IBS (Mujagic et al., 2016). A novel multi-domain non-invasive biomarker panel was identified and validated, which could discriminate IBS from health with high sensitivity (88.1%) and specificity (86.5%), and could be correlated with GI symptom severity in IBS and in the general population (Mujagic et al., 2016). This included plasma cytokine levels, such as IL-1β, IL-6, IL12p70, and TNF-α, as markers of systemic immune activation, fecal Chromogranin A (CgA), as an indicator of the colonic neuroendocrine cell activity, fecal human β-defensin 2, as an indicator of host protection against microbes, calprotectin, as an indicator of colorectal inflammation, reflecting neutrophil migration to the colonic mucosa, and caproate, a product of microbial fermentation of non-digested oligosaccharides in the colon.

Recent studies have highlighted the role of immune dysregulation and microbial dysbiosis in IBS and some molecules of the immune system measured in blood or in GI luminal contents could be putative biomarkers. Fecal CgA plays a role in pain regulation and antimicrobial activity, and their fecal levels have been negatively correlated with colonic transit time in individuals with IBS (Öhman et al., 2012). The fecal granin profile of IBS has been associated with the microbiota alpha-diversity and composition, in particular with the genus *Bacteroides* (Sundin et al., 2018). Although CgA represents a link between the neuroendocrine and immune systems, fecal and serum granins can be increased in other conditions, including lymphocytic colitis (El-Salhy et al., 2011) and celiac disease (Pietroletti et al., 1986). Granins thus are not considered as useful biomarkers for IBS, because their lack of specificity and discriminatory power.

Calprotectin, a protein released by neutrophils during GI inflammation, can be easily measured in stool samples, as it is resistant to degradation in the colon, and can therefore be considered as a non-invasive marker of low-grade inflammation. Although calprotectin is primarily used to distinguish IBS from IBD (Chang et al., 2014; Banerjee et al., 2015), concentrations have been shown to vary within IBS. In a prospective study, fecal calprotectin was elevated in one third of all patients across IBS subtypes (Melchior et al., 2017). In addition, a recent study demonstrated that differences in fecal calprotectin concentrations in children discriminated between IBS subtypes and from healthy controls. In particular, fecal calprotectin concentration was highest in IBS-D, followed by those with IBS-M and IBS-C (Choi and Jeong, 2019). In combination with other plasma and fecal biomarkers, fecal calprotectin may effectively discriminate IBS from health and within IBS subtypes (Nemakayala and Cash, 2019).

Serine proteases, such as tryptases, which are released by colonic mast cells and bacteria, have been also reported to be elevated in IBS-D (Róka et al., 2007; Tooth et al., 2014). These proteases are thought to play a role in several pathways involved in IBS symptom generation, such as the stimulation of colonic nerves through the protease activated receptor-2, leading to abdominal pain (Valdez-Morales et al., 2013; Cattaruzza et al., 2014). Proteases also contribute to mucosal inflammation (Róka et al., 2007), affect motility of smooth muscles (Sekiguchi et al., 2006) and increase paracellular permeability (Róka et al., 2007) in the colon.

TLRs are a family of receptors present on both epithelial and immune cells in those tissues exposed to the external environment, such as the lungs and GI tract (Zarember and Godowski, 2002). Alterations in the activation of TLR1/2, TLR2, TLR3, TLR5, TLR7, and TLR8 have been reported in IBS (Brint et al., 2010), such as increased levels of TLRs 4/5 (Zarember and Godowski, 2002; Shukla et al., 2018) and decreased levels of TLRs 7/8 (Brint et al., 2010; Clarke et al., 2012). TLRs bind to conserved microbial molecular patterns and their activation induces intracellular signaling cascades leading to the expression of pro- and anti-inflammatory cytokines and chemokines (Vidya et al., 2018). In addition, it has been demonstrated that TLR activation can have consequences on colonic motility, through the activation of neuroendocrine mechanisms (Tattoli et al., 2012; Shukla et al., 2018), or through interactions with the sulfide system (Grasa et al., 2019). In particular, TLR4 seems to play a crucial role in the maintenance of normal colonic motility, as Tlr4^−/−^ mice showed a decreased amplitude and frequency of the contractions in the proximal colon (Forcén et al., 2016). In human primary cultures of colonic smooth muscle cells, lipopolysaccharide-induced TLR4 activation resulted in an increased myogenic effect, whereas the incubation with TLR2 agonists induced a decreased myogenic effect (Tattoli et al., 2012).

**Figure 6:**
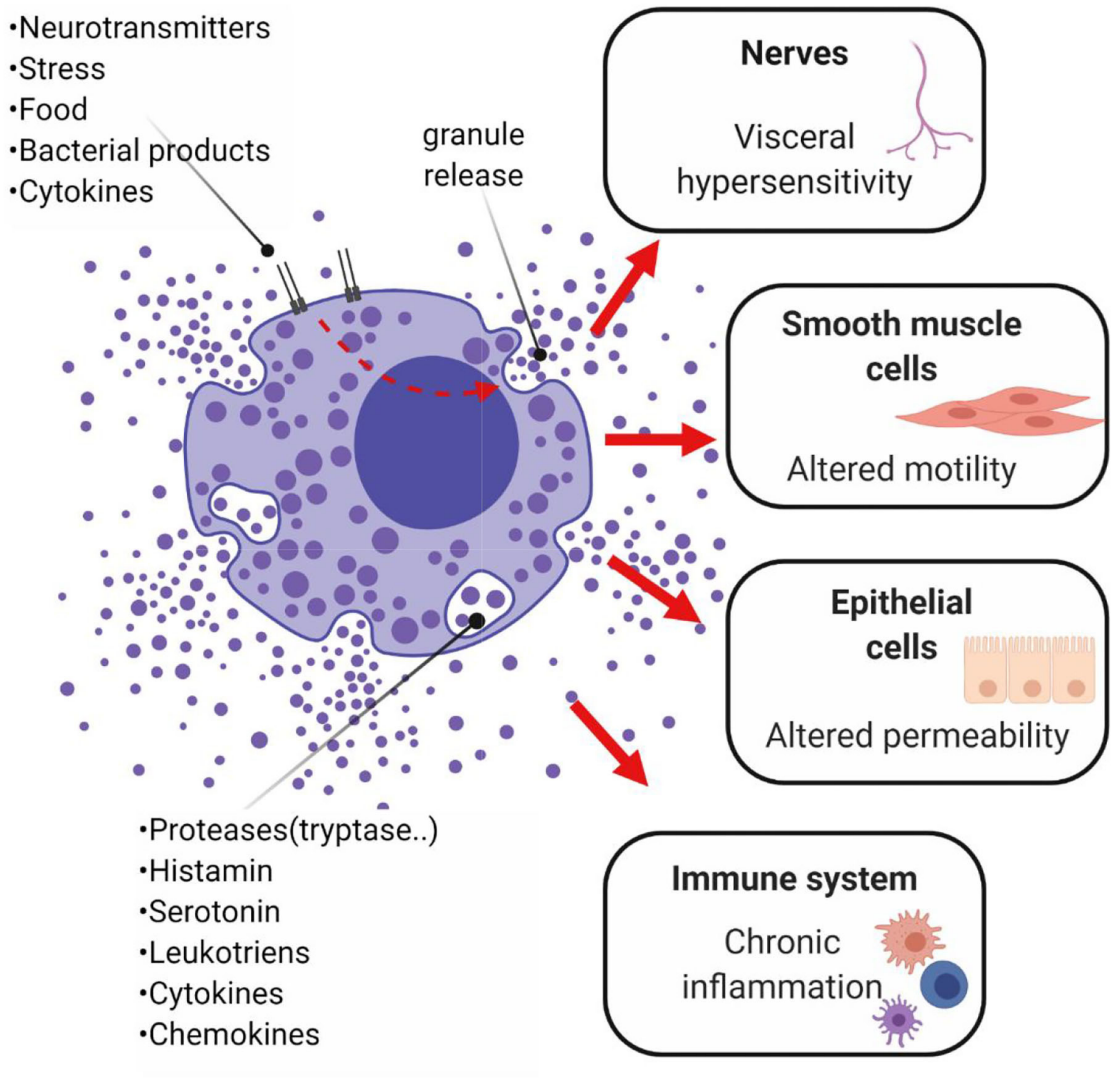
Potential role of mast cells in IBS and chronic low-grade inflammation. Mast cells are thought to play a role in the onset of abdominal pain, as well as diarrhea or constipation. These symptoms are modulated by the mediators released by activated mast cells of the GI mucosa, which stimulate other immune cells, perpetuate chronic inflammation and alter secretion and peristalsis, resulting in abnormal GI permeability and motility. Mast cells, located close to nerve fibers, are thought to trigger pain signals. The mediator histamine sensitizes the nociceptor transient receptor potential channel V1 on peripheral nerve terminal of nociceptive submucosal neurons, resulting in visceral hypersensitivity (Cenac et al., 2010). Studies on rectal biopsies from IBS subjects demonstrated that the histamine H1 receptor-mediated stimulation of the nociceptor transient receptor potential channel V1 was potentiated in IBS subjects but not in healthy controls (Wouters et al., 2016). Proteases degranulated by mast cells may also destroy various epithelial gap junctional proteins (e.g., zonula occludens), leading to impairments in epithelial barrier function. Alterations in motility seem also to be linked to mast cells' degranulation. In particular, the stimulation of prostanoid receptors P2X on smooth muscle cells generates the excitatory potential responsible for contraction, impacting on smooth muscle contractility (Zhang L. et al., 2016). Created with BioRender.com.

Consistent with these observations, chronic low-grade inflammation and differences in pro- and anti-inflammatory cytokine concentrations in the colonic mucosa or systemically have been also associated with IBS (Sundin et al., 2015; Choghakhori et al., 2017). Several studies report an increase in the concentration of pro-inflammatory cytokines, such as IL-1β, IL-6, IL-8, TNF-α and IFN-γ (Dinan et al., 2006; Rana et al., 2012; Darkoh et al., 2014; Barbaro et al., 2016; Seyedmirzaee et al., 2016; Choghakhori et al., 2017; Bennet et al., 2018; Vara et al., 2018), and a decrease in the concentration of the anti-inflammatory cytokine IL-10 (Macsharry et al., 2008; Choghakhori et al., 2017) in serum, plasma or colonic biopsies of IBS patients. However, these changes are inconsistent between studies (Chang et al., 2012; Shulman et al., 2014). Differences in the count and the activation rate of immune cell populations, particularly mast cells but also macrophages, lymphocytes and eosinophils, have also been reported in IBS (Lee et al., 2008; Walker et al., 2009). Mediators produced by these cells (nitric oxide, histamine and proteases) are likely to play a role in several pathways involved in IBS symptoms generation (Figure 6). Notably, the number and the activation rate of mucosal mast cells has been reported to be higher in IBS-D patients compared to healthy controls and correlated with severity and frequency of abdominal pain (Barbara et al., 2004; Park et al., 2006). Another study reported no difference in mast cell count, but the percentage of degranulated mast cells was increased in IBS-D patients (Liu et al., 2018). In addition to the number of colonic mast cells, an augmented activity of colonic mast cells in proximity to sensory nerves is likely to play a role in IBS symptom development. In subjects with IBS-D the immune activation of peripheral CD4+ T-cells was reported, but it did not correlate with GI or psychological symptoms (Nasser et al., 2019), whereas an enhanced pro-inflammatory cytokine release in IBS-D was associated with symptoms and anxiety in a previous study (Liebregts et al., 2007).

An increased count of lamina propria CD3^+^, CD4^+^, and CD8^+^ T cells and activated macrophages has been observed also in subjects with a diarrhea-predominant phenotype persisting after an episode of infectious gastroenteritis (Spiller et al., 2000). In post-infectious IBS, the initial infection may have altered the normal GI microbial environment and led to a prolonged immune response (Al-Khatib and Lin, 2009), persisting even when the infecting pathogen was no longer detectable (Spiller et al., 2000). The cytolethal distending toxin, produced by gram-negative pathogenic bacteria which often persistently colonize their host, together with the cytoskeletal protein vinculin, have been recently used as biomarkers to successfully discriminate IBS-D from other causes of diarrhea and healthy controls (Rezaie et al., 2017), advancing the understanding of the role of immunity in FGIDs, although the diagnostic value of these biomarkers is less certain (Talley et al., 2019).

## Conclusions

FGIDs present a highly variable clinical phenotype associated with early childhood events, somatisation, different diets, psychological, hereditary and environmental factors. To date, specific immune cell populations, cytokine concentrations and bioactive metabolites have been investigated in an independent manner, resulting in contrasting findings on the exact role of immune activation in the development of FGIDs (Lazaridis and Germanidis, 2018).

Several studies have provided new insights into bacterial mechanisms influencing the immune system in the context of inflammatory bowel diseases (Gonçalves et al., 2018), but less is known about IBS (Barbara et al., 2011).

The evaluation of the consequences of dysbiosis in FGIDs has some limitations. Firstly, there is still a lack of integration between taxonomic and functional data for the identification of specific microbes and to better understand their contribution to the optimal function of the GI tract and associated organs, for example the brain via the gut-brain axis. Indeed, the interactions between microbial community and host could not be gathered from single analyses as most metabolic pathways in nature take place within communities, rather than pure cultures. High-throughput DNA sequencing technology has enabled a shift from descriptive analysis of different taxa of the microbial community to an investigation of the predictive functional contribution of the microbiota to health and disease (Rooks and Garrett, 2016).

Secondly, IBS clinical symptoms are heterogenous and fluctuating and there are no confirmed molecular or organic biomarkers to diagnose this condition. Finally, the identification of a microbial signature in IBS is confounded by the individual complexity, instability and variability of the microbiota, which can be influenced by the psychological status, medications and diet. In this regard, diet can affect microbiota composition and function as well as colonic motility, sensitivity and epithelial barrier function. However, further research is needed to elucidate the role of specific macronutrients and micronutrients in IBS.

Finally, discrepancies between studies may also reflect differences in DNA extraction methods, analytic techniques, number of subjects and the sample collection method. Indeed, fecal samples do not precisely represent the microbiota composition or function in the proximal colon, and colonic biopsies do not physiologically reflect the microbiota, because of the extensive sample preparation.

A possible microbial pathogenesis in IBS has also therapeutic implications. In this regard, probiotic, prebiotic, synbiotic and antibiotic treatments have been largely investigated although with contrasting results, and the manipulation of GI microbiota represents a promising strategy in the treatment of FGIDs.

In this review, the recent evidence proposing FGIDs as systemic conditions has been discussed. This involves not only individual systems, such as the GI microbiota, the digestive, immune and enteric nervous systems, but also their intricate interplay. The mechanisms involved in FGID pathophysiology can be investigated at the cellular and molecular level, including the analysis of the genome, trascriptome, proteome, metabolome and brain connectome. Therefore, we suggest an integrative system biology approach as the most appropriate to investigate the complex interactions underlying FGIDs, considering the broad range of different and interacting elements, which are responsible for the highly variable clinical phenotype.

## Author Contributions

CC prepared the first draft and WY, RG, NT, WM, and NR edited and approved the final manuscript. All authors contributed to the article and approved the submitted version.

## Conflict of Interest

The authors declare that the research was conducted in the absence of any commercial or financial relationships that could be construed as a potential conflict of interest.

## Abbreviations

BCFAs: branched-chain fatty acids
CgA: Chromogranin A
FC: Functional Constipation
FD: Functional Diarrhea
FGIDs: Functional Gastrointestinal Diseases
FODMAPs, Fermentable Oligosaccharides, Disaccharides: Monosaccharides And Polyols
GI: Gastrointestinal
GPRs: G-protein-coupled receptors
HDAC: histone deacetylases
IBS: Irritable Bowel Syndrome
IBS-C: IBS-Constipation
IBS-D: IBS-Diarrhea
IBS-M: IBS-Mixed
IBS-U: IBS-Unclassified
IFN: Interferon
IL: Interleukin
SCFAs: Short Chain Fatty Acids
SRB: sulfate-reducing bacteria
TJs: Tight Junctions
TLR: Toll-like Receptor
TNF: Tumor Necrosis Factor.
